# *Mage* transposon: a novel gene delivery system for mammalian cells

**DOI:** 10.1093/nar/gkae048

**Published:** 2024-02-01

**Authors:** Jinghan Tian, Doudou Tong, Zhendong Li, Erqiang Wang, Yifei Yu, Hangya Lv, Zhendan Hu, Fang Sun, Guoping Wang, Min He, Tian Xia

**Affiliations:** Institute of Pathology, Department of Pathology, School of Basic Medicine, Tongji Hospital, Tongji Medical College, Huazhong University of Science and Technology, Wuhan, Hubei 430030, China; Institute of Pathology, Department of Pathology, School of Basic Medicine, Tongji Hospital, Tongji Medical College, Huazhong University of Science and Technology, Wuhan, Hubei 430030, China; Elongevity Inc, Wuhan, Hubei 430000, China; Institute of Pathology, Department of Pathology, School of Basic Medicine, Tongji Hospital, Tongji Medical College, Huazhong University of Science and Technology, Wuhan, Hubei 430030, China; Elongevity Inc, Wuhan, Hubei 430000, China; Elongevity Inc, Wuhan, Hubei 430000, China; Elongevity Inc, Wuhan, Hubei 430000, China; Elongevity Inc, Wuhan, Hubei 430000, China; Institute of Pathology, Department of Pathology, School of Basic Medicine, Tongji Hospital, Tongji Medical College, Huazhong University of Science and Technology, Wuhan, Hubei 430030, China; Elongevity Inc, Wuhan, Hubei 430000, China; Institute of Pathology, Department of Pathology, School of Basic Medicine, Tongji Hospital, Tongji Medical College, Huazhong University of Science and Technology, Wuhan, Hubei 430030, China; School of Artificial Intelligence and Automation, Huazhong University of Science and Technology, Wuhan, Hubei 430074, China

## Abstract

Transposons, as non-viral integration vectors, provide a secure and efficient method for stable gene delivery. In this study, we have discovered *Mage* (*MG*), a novel member of the *piggyBac*(*PB*) family, which exhibits strong transposability in a variety of mammalian cells and primary T cells. The wild-type *MG* showed a weaker insertion preference for near genes, transcription start sites (TSS), CpG islands, and DNaseI hypersensitive sites in comparison to *PB*, approaching the random insertion pattern. Utilizing *in silico* virtual screening and feasible combinatorial mutagenesis *in vitro*, we effectively produced the hyperactive *MG* transposase (hyMagease). This variant boasts a transposition rate 60% greater than its native counterpart without significantly altering its insertion pattern. Furthermore, we applied the hyMagease to efficiently deliver chimeric antigen receptor (CAR) into T cells, leading to stable high-level expression and inducing significant anti-tumor effects both *in vitro* and in xenograft mice models. These findings provide a compelling tool for gene transfer research, emphasizing its potential and prospects in the domains of genetic engineering and gene therapy.

## Graphical Abstract

**Graphical Abstract:**
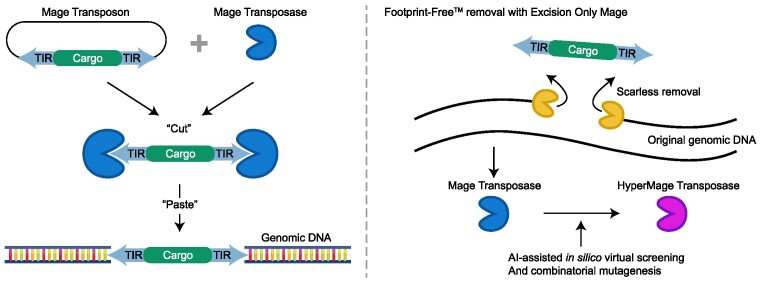

## Introduction

Transposons, a class of genetic elements capable of migrating and replicating within the host genome, exhibit a fascinating mechanism known as transposition, wherein they change their insertion site. These elements can be broadly categorized into DNA transposons and retrotransposons (1,2). DNA transposons are mobilized through a ‘cut-and-paste’ mechanism, as they are excised from their original location and integrated into a new target site within the genome. This movement is facilitated by the transposase enzyme recognizing end repeats. The first DNA transposon was discovered in maize by Barbara McClintock (3), and subsequent research revealed that these elements are active in a wide range of organisms (4–7). Within mammalian cells, three prominent DNA transposons are commonly employed: *Sleeping Beauty* (*SB*) from the *Tcl/mariner* superfamily (8), *piggyBac* (*PB*) from the *piggyBac* superfamily (9), and *Tol2* from the *hAT* superfamily (10). Moreover, numerous active transposons from various superfamilies have been recently discovered, including *TcBuster* (11) and *Tgf2* (12) from the *hAT* superfamily, *ZB* (13) from the *Tcl/mariner* superfamily, and *Passer* from the *pogo* superfamily (14), among others. These DNA transposons have proven invaluable in various genetic studies, providing insights into genome engineering, gene therapy, and understanding the mechanisms underlying transposition events.

Among the widely used transposons mentioned above, *PB* exhibits the highest transposable activity in human cell lines and primary T cells (15,16). During its transposition, *PB* specifically integrates into the TTAA site, ensuring a clean excision without leaving residues (9,17). Its unique genetic engineering traits make *PB* a popular choice in fields such as gene therapy (18–20), gene trapping (21), gene transfer (22,23), transgenic animals (24), gene screening (25–27), and more. In addition to the founder *PB* transposable elements, the *PB* superfamily comprises two major types of elements: *piggyBac*-like elements (PLE) and *piggyBac*-derived sequences (PGBD). *PB* and PLE share characteristics, including a TTAA tandem site repeat (TSD), a triple aspartate (DDD) pattern, and the C-terminal cysteine-rich structural domain (CRD) in the *PB* transposase (7,28). Some PLEs have recently been identified in insect species and baculoviruses (7,29–35).

Transposase and terminal inverted repeats (TIRs) are naturally found together in transposons. It is possible to create a two-component transposon system where the gene of interest is positioned between transposon TIRs, and transposition is facilitated by transposase expression (36). Such designs have paved the way for the development and utilization of transposable molecular vehicles in various organisms. The key factors for transposon application in genetic engineering are transposition efficiency and preference. Manipulation of transposon TIRs or transposase proteins can enhance transposition efficiency. Hyperactive SB vectors, for instance, have been generated by shortening the TIRs and introducing specific mutations (37,38). The effective application of modified transposase proteins has been demonstrated in both the *SB* and *PB* transposon systems. Notable examples include the development of high-activity variants of *SB* transposases, such as the remarkably active hyper *Sleeping Beauty* 100X (hySB100X) (39) and the high-solubility *SB* transposase (hsSB) (40), achieved through distinct *in silico* approaches. Additionally, high-performing transposases like hyperactive PBase (hyPB) (41) and bz-hyPBase (42) were identified through yeast-based screening. Transposons exhibit diverse insertion patterns at the genome-wide level, which influences their applications. *PB* and *Tol2* transposons share a preference for inserting near transcription start sites (TSSs), DNaseI hypersensitive sites, and CpG islands (16). In contrast, the insertion of *SB* transposons is closer to random (43–45).

Transposons, as controllable DNA carriers, have a broad range of applications in cell and gene therapy, particularly in the revolutionary CAR-T cell therapy. While many approaches require viral vectors to achieve stable expression of CAR T cells, non-viral vector DNA transposons have emerged as a popular alternative due to their cost-effectiveness and reduced risk of viral residues (46). CAR-T cells modified with non-viral transposon vectors have demonstrated promising efficacy in preclinical and early clinical trials. For instance, *SB* vectors with anti-CD19 have been successful in generating functional CAR T cells (47,48) and have shown safety in adjuvant therapy for patients with B cell-Acute Lymphoblastic Leukemia (B-ALL) and non-Hodgkin's Lymphoma (NHL) (49). *PB* vectors have also been utilized to produce CD19 CAR T cells for treating B-cell ALL and NHL (50). Moreover, CD19 CAR T cells generated using the *piggyBat* transposon from the *PB* superfamily demonstrate specific cytotoxic efficacy both *in vivo* and *in vitro* (51).

In a word, there is an urgent need to develop a DNA transposon that combines high transposition efficiency with safety. In our research, we have identified a novel element, *MG*, from the *piggyBac* superfamily, which demonstrates efficient genetic modification in various cell lines and primary T cells. We further model the wild-type (wt) *MG* transposase protein *in silico* and employed reasonable amino acid substitutions and implemented feasible combinatorial mutations to generate hyperactive transposase variants. Using the hyperactive *MG* transposase variant, we successfully transfected NKG2D CAR into primary T cells, resulting in specific targeting of tumor antigens and effective killing effects in mice with severe immunodeficiency.

## Materials and methods

### Genome searches

Sequence searches were conducted to identify *piggyBac*-like elements (PLE) in the genomes of Lepidoptera species. *PB* transposons were queried using BLAST (52) to detect coding sequences related to all known PLE transposons in RepBase and GenBank. The top 10–30 nonoverlapping hits (*E*-value <10^−5^) were extracted, along with 500 bp of flanking sequence, and aligned using the ClustalW (53) program. For each filtered transposon, coding sequences were predicted using Open Reading Frame (ORF) Finder (https://www.ncbi.nlm.nih.gov/orffinder/), and domains were predicted using HMM hmmscan (54). Transposons with a ‘DDE_Tnp_1_7’ domain, domain score >150, and domain length >275 were selected as candidates.

### Evolutionary relationships

The evolutionary history was inferred using the Neighbor-Joining method (NJ) (55). The bootstrap consensus tree (56), based on 1000 replicates, is presented to represent the evolutionary history of the analyzed taxa. Branches corresponding to partitions reproduced in less than 50% of bootstrap replicates are collapsed. The percentage of replicate trees in which the associated taxa clustered together in the bootstrap test (1000 replicates) is indicated next to the branches. Evolutionary distances were computed using the Poisson correction method (57) and are expressed as the number of amino acid substitutions per site. This analysis involved 33 amino acid sequences. All ambiguous positions were removed for each sequence pair (pairwise deletion option). The final dataset comprised a total of 975 positions. Evolutionary analyses were performed using MEGA X (58).

### Structure modeling

For modeling the *MG* transposase protein (Magease), we employed the AlphaFold v2.0 algorithm (59), a deep neural network-based end-to-end modeling tool that generates structural models from sequence data. Raw multiple sequence alignments (MSA) for each chain were prepared using the published AlphaFold pipeline (59), querying full databases such as UniRef90 version 2020_01, MGnify version 2018_12, Uniclust30 version 2018_08, and BFD. To model the TIR DNA, we utilized the Avogadro v1.2 (60) software to construct the B-DNA conformation, which is the predominant structural form of DNA in cells. Subsequently, we employed Haddock v2.4 (61), an advanced biomolecular docking software driven by high ambiguity, to generate protein-DNA docking complexes using the best-predicted protein and DNA models. The AlphaFold was run in the environment with Ubuntu 18.04.6 LTS and CUDA Version 11.4. Haddock was executed on a webserver (https://wenmr.science.uu.nl/haddock2.4/).

Before performing *in silico* docking with Haddock, Ambiguous Interaction Restraints (AIR) were required. These AIRs were created between each active residue of one partner and the combination of active and passive residues of the other partner. Specifically, active residues on the *MG* protein were taken from CPORT (62) predictions, while all DNA nucleotides were defined as passive. The final docking results were ranked by the HADDOCK (61) score, calculated as follows: HADDOCKscore = 1.0 * Evdw + 0.2 * Elec + 1.0 * Edesol + 0.1 * Eair (where Evdw represents the intermolecular van der Waals energy, Elec is the intermolecular electrostatic energy, Edesol represents an empirical desolvation energy term, and Eair is the AIR energy). After performing Root-Mean Square Deviation (RMSD) clustering with a cutoff of 5Å on the docking results, we considered the top 4 members of each cluster as valid candidates for the docking. The resulting docking structure with the highest HADDOCKscore was selected as the representative *MG* protein and DNA binding complex, as shown in Figure 4.

### Plasmid construction

The backbone plasmid for kanamycin resistance was obtained from pMAX-cloning (Lonza, Switzerland), with *SB* transposon element TIR sequences from pT2/BH (Plasmid #26556, Addgene). The human elongation factor-1α promoter was used as a promoter (EF1α_mCherry_P2A_Hygro_Barcode, plasmid #120426, Addgene) to initiate the enhanced green fluorescence protein (EGFP) or puromycin resistance cassette. Transposase and promoter sequences from pCMV (CAT) T7-SB100 (Plasmid #34879, Addgene). These sequences were synthesized (GenScript, USA) to create pMAX-SB/Ef1α-EGFP, pMAX-SB/Ef1α-puromycin, and pCMV-SB100X. The hyperactive version of *SB*, pCMV-hySB100X, was constructed using The Mut Express II Fast Mutagenesis Kit V2 (Vazyme, China).

The *PB* and *MG* transposon vector were constructed as follows. For example, 5′TIR and 3′TIR of *PB* and *MG* sequences were digested with BsaI-EcoRI and EcoRI-BsaI respectively and ligated to BsaI fragment of pMAX-SB/Ef1α-EGFP, resulting in pMAX-PB and pMAX-MG. To construct the transposon vector pMAX-PB/Ef1α-EGFP and pMAX-MG/Ef1α-EGFP, the BamHI-NsiI fragment containing the EGFP was released from pMAX-SB/Ef1α-EGFP and cloned into the BamHI-NsiI sites of pMAX-PB or pMAX-MG. To construct the transposon vector pMAX-PB/Ef1α-puromycin and pMAX-MG/Ef1α-puromycin, the internal ribosome entry site (IRES) followed by a puromycin resistance cassette cloned into pMAX-SB/Ef1α-EGFP and pMAX-PB/EF1α-EGFP backbone plasmids with EcoRI-SalI restriction sites through EcoRI-XhoI. To create hyperactive *PB* and *MG* transposase expression vector pCMV-hyPB and pCMV-MG, the hyperactive *PB* and *MG* transposase-containing fragments were released by AgeI-SalI digestion and cloned into the AgeI-SalI site of pCMV-SB100X respectively, resulting in pCMV-hyPB and pCMV-MG.

The NKG2D CAR vector consists of the extracellular structure of human NKG2D (NKG2D ED; Uniprot: P26718-1; aa 83–216), the IgG4 hinge region, the CD28 transmembrane region, the DAP12 signaling motif, and the intracellular stimulatory structure of 4–1BB. The control CAR is a replacement of NKG2D with anti-CD19 scFv. The NKG2D CAR sequence was custom synthesized and cloned into MG donor plasmids by EcoRI-SalI, respectively.

The sequences of *PB* TIRs, *MG* TIRs, hyperactive *PB* transposase-coding sequence, *MG* transposase-coding sequence, NKG2D CAR, and CD19 CAR sequence were synthesized (GenScript, USA) (*MG* relevant sequences shown in Supplementary Table S1).

### Colony formation

A total of 6 × 10^4^ HeLa cells were co-transfected with a donor plasmid (50 or 250 ng) containing the puromycin resistance gene and a helper DNA plasmid (ranging from 0 to 1000 ng) using Lipofectamine 3000 (Thermo Fisher Scientific, USA). Twenty-four hours post-transfection, the cells were trypsinized and seeded on 10 cm plates for stable integration of transposons (for 250 ng of donor plasmid, 3 × 10^3^ of the transfected cells were utilized, and for 50 ng of donor plasmid, 4 × 10^5^ of the transfected cells were employed). The selection was performed with 0.4 μg/ml puromycin (Beyotime Biotechnology, China). After a fortnight of selection, the surviving colonies were fixed with 10% formalin, stained with crystal violet, and subsequently counted.

### Transposition assays

To evaluate the transposition efficiency of various transposon systems and different mutant *MG* transposases in Jurkat cells, we employed electroporation as the method of choice. In one experiment designed to gauge the transposition activity of different transposon systems, a total of 8 × 10^6^ Jurkat cells (ATCC, USA) were subjected to electroporation with 4 μg of transposon (pMAX-MG/EF1α-GFP, pMAX-SB/EF1α-GFP, or pMAX-PB/EF1α-GFP) along with 4 μg of transposase (pCMV-MG, pCMV-hyMG, pCMV-hySB100X, or pCMV-hyPB). In the experiment measuring the transposition efficiency of different mutant *MG* transposases, 8 × 10^6^ Jurkat cells (ATCC, USA) underwent electroporation with 4 μg of transposon (pMAX-MG/EF1α-GFP) and 4 μg of various mutant *MG* transposases. Electric pulses for both experiments were delivered using a BTX Electro Square Porator EM830 (Harvard Apparatus, USA). Post-electroporation, cells were cultured in RPMI 1640 medium supplemented with 10% fetal bovine serum (Thermo Fisher Scientific, USA) for a span of 14 days. At least three independent experiments were conducted to compare the relative transposition efficiencies *in vitro*. For evaluating transposition efficiency in T cells, TransAct (Miltenyi Biotech, Germany) was employed to stimulate T cells derived from cryopreserved peripheral blood mononuclear cells using AIM-V medium (Life Technologies, USA), supplemented with 5% fetal bovine serum (Gibco, USA) and 300 IU/ml interleukin-2 (R&D Systems, USA). Following this, 8 × 10^6^ T cells were transfected with 4μg of pMAX-MG/EF1α-GFP and 4μg of pCMV-MG using the P3 Primary Cell 4D Nucleofector Kit (Lonza, Switzerland) on a 4D Nucleofector device. The next day, transfected cells were analyzed for GFP expression by flow cytometry (ACEA Biosciences, USA).

### Flow cytometry

The flow cytometry data were analyzed using FlowJo vX.0.7 software (Beckman Coulter, USA). To assess the transposition efficiency of the EGFP expression transposon, 5 × 10^5^ cells were sampled after 14 days of cell culture and subjected to analysis for green fluorescent protein (GFP) expression using the FITC channel. The expression of CAR T cell NKG2D was determined by detecting the Strep II tag (GenScript, USA).

### Real-time PCR of transposon copy number

GFP^+^ T cells and NKG2D CAR T cells extracted genomic DNA (Vazyme, China). Dpn I restriction enzyme (Thermo Fisher Scientific, USA) was utilized to digest the genomic DNA and remove any residual unintegrated plasmid. The AceQ qPCR SYBR Green Master Mix (Vazyme, China) was employed for qPCR analysis. To determine the average number of integrated MG-transposons, specific primers targeting the EGFP reporter gene, q-EGFP-F (AGAACGGCATCAAGGTGAAC) and q-EGFP-R (TGCTCAGGTAGTGGTTGTCG), were used. Standard curves were established by performing serial dilutions of the respective transposon plasmids with known copy numbers (Supplementary Figure S1A). Similarly, to measure the average copy number of transposons integrating the NKG2D CAR into cells, a set of primers targeting the NKG2D gene, q-NK-F (ACTTCCTGGGACGACTTGTT) and q-NK-R (TGGCCCTGCAGTTCTTGATA), were used. Standard curves were generated using serial dilutions of the respective transposon plasmids with known copy numbers (Supplementary Figure S1C). All samples were normalized against genomic RNaseP copies, which typically exist at 2 copies per human cell genome. The RNaseP copy number was determined using RPF (AGATTTGGACCTGCGAGCG) and RPR (GAGCGGCTGTCTCCACAAGT) primers and a standard curve derived from seven logarithmic dilutions of a plasmid carrying each target gene (Supplementary Figure S1B).

Standard curves were established using serial dilutions of known transposon plasmids. The same methodology was adopted for the NKG2D CAR. All samples were normalized against genomic RNaseP copies, which typically exist at 2 copies per human cell genome. RNaseP copy number was ascertained using specific primers and a standard curve.

### Cytotoxicity assay

HeLa cells were transfected with varying concentrations of the transposon donor plasmid, helper plasmid, and pMAX-cloning (Lonza, Switzerland) to investigate any potential cytotoxic effects induced by the *MG* transposon. Cell viability was assessed using the Cell Counting Kit-8 (MCE, USA). Approximately 5 × 10^3^ HeLa cells were seeded per well in a 96-well plate one day prior to transfection. Transfection of plasmids into HeLa cells was performed using Lipofectamine 3000 (Thermo Fisher Scientific, USA). After 3 days, the cells were treated with a Cell Counting Kit-8 solution, and the relative cell viability was determined by measuring the absorbance at 490 nm using a Microplate Absorbance Reader (Tecan, Switzerland).

To detect the cytolytic activity of CAR-modified T cells, a non-radioactive method was employed, specifically the DELFIA EuTDA Cytotoxicity Reagents kit (PerkinElmer, USA).

### Excision assay

The transfection was performed using Lipo3000 transfection reagent (Thermo Fisher Scientific, USA). A total of 3 × 10^5^ HeLa cells were transfected with 500 ng of the donor plasmid containing the puromycin resistance gene (pMAX-MG/EF1α-GFP) and 500 ng of the helper plasmid (pCMV-MG). After two days of transfection, the genome was extracted using the FastPure Cell/Tissue DNA Isolation Mini Kit (Vazyme, China). Approximately 500 ng of the genome was used for PCR, and the PCR primers were designed on the vector backbone outside of *MG* TIR. Following PCR amplification, the PCR products were cloned into the pMD20-T vector (TAKARA, Japan). The footprint resulting from transposon excision was identified through sequencing (TsingKe, China).

### Constructing and sequencing a library of integration sites

Genomic DNA from CAR-modified T cells was extracted using the FastPure Cell/Tissue DNA Isolation Mini Kit (Vazyme, China). Enhanced-specificity Tag-PCR was conducted following the published protocol (63). In brief, tagmentation was performed using 500 ng of gDNA and the Illumina DNA prep kit (Illumina, USA). Subsequently, the first-round PCR and the second nested PCR were carried out using KAPA HiFi HotStart ReadyMix (KAPA Biosystems, USA). The primer sets for the two rounds of PCR are listed in Supplementary Table S2, and the primer pairing information can be found in Supplementary Table S3. The PCR products were size-selected using KAPA Pure Beads (KAPA Biosystems, USA). Purified samples were quantified using a Qubit Fluorometer (Thermo Fisher Scientific, USA). The sequence libraries generated from Tag-PCR were sequenced on a Nova6000 next sequencing platform with paired-end 250 bp reads (Illumina, USA).

### Bioinformatics analysis of integration sites

Reads passing the quality filter were aligned to the hg38 human genome using STAR, then IntegrationSiteMapper of DISCVR-seq Toolkit was used to obtain the integration sites (Reads mapped to a unique site >10, the minimum MAPQ to consider an alignment is 20). For assessing the site of genomic integration, we examined the presence of genes, CpG islands, transcription start sites (TSSs), and DNase I hypersensitivity sites. The Cancer gene list was obtained from the COSMIC cancer database. To evaluate the frequency of integration into genomic safe harbors (GSHs), we employed previously established criteria. These criteria include (i) A minimum distance of 50 kb from the 5′ end of any gene, (ii) A minimum distance of 300 kb from any cancer-related gene, (iii) A minimum distance of 300 kb from any microRNA (miRNA), (iv) Locations outside of transcription units, (v) Locations outside of ultraconserved regions of the human genome. To establish a baseline for comparison, we simulated random integration sites by generating random numbers across the hg38 coordinate system.

### Transposase engineering

For each HADDOCK-predicted candidate MG-DNA docking structure, we identified the interface residues using the ‘interface residues’ script in PyMOL (64) with a cutoff of 1 Å. We considered non-conserved domain residues as interfaces between the protein and DNA if they were shown as interfaces in more than two-thirds of all the docking structures. These residues were defined as our target mutation points. Subsequently, we explored three distinct mutation options for each mutation point: (i) converting hydrophobic amino acids to hydrophilic amino acids, (ii) substituting acidic amino acids with basic amino acids, and (iii) incorporating naturally occurring sequence variants into the transposase.

### Site-directed mutagenesis of the MG transposase

The Mut Express II Fast Mutagenesis Kit V2 (Vazyme, China) was utilized for constructing the Magease targeted mutations. The ‘CE Design’ software provided primer design guidelines (https://www.vazyme.com/companyfile/7/). To confirm the presence of the desired mutation, individual clones on LB plates were purified using the QIAprep spin miniprep kit (QIAGEN, Germany) and subjected to Sanger sequencing (TsingKe, China).

### Preparation and expansion of CAR-T cells

Peripheral blood mononuclear cells (PBMCs) used in this study were purchased from Milestone Biotechnologies. To obtain αβ T cells, human PBMCs were activated with TransAct (Miltenyi Biotech, Germany) and cultured in AIM-V medium (Life Technologies, USA) supplemented with 5% fetal bovine serum (Gibco, USA) and 300 IU/ml interleukin-2 (R&D Systems, USA). After 3–5 days of incubation, αβ T cells were harvested. A total of 1 × 10^7^ cells were electroporated with 5 μg of transposase and 10 μg of NKG2D CAR/CD19 CAR donor plasmid. The electroporation was performed using the EO-115 program of the 4D-Nucleofector system (Lonza, USA). Subsequent to electroporation, feeder K562 cells expressing the CD64, CD86, and CD137L markers (hereafter referred to as K562A) were irradiated with 50 Gy γ radiation (Blood Center, Wuhan, China) and co-cultured with CAR-T cells (65). In AIM-V medium supplemented with 5% fetal bovine serum and 300 IU/ml IL-2, the electroporated T cells and feeder cells were seeded at a 1:2 ratio and cultured for 9–11 days. The culture medium was renewed every 2–3 days.

To expand non-modified T cells or electroporated CD19 CAR T cells, they were co-cultured with γ-irradiated K562A cells at a ratio of 1:50. Additionally, 60 ng/ml of OKT3 was added at the start of co-culture and replaced with 300 IU/ml of IL-2 medium at 2–3 days intervals.

### Xenograft mouse model

The Animal Care and Use Committee of Huazhong University of Science and Technology approved all animal experiments. Female NCG mice aged 6–8 weeks were intraperitoneally injected with 2 × 10^6^ firefly-luciferase-expressing HCT116 tumor cells to establish tumor models. Tumor-bearing mice were treated with intraperitoneal injections of 1 × 10^7^ NKG2D CAR-modified T cells. Unmodified T cells or PBS-treated mice were used as controls. Tumor progression was assessed using bioluminescence imaging. Mice were anesthetized with isoflurane and administered 0.3 mg of d-luciferin per gram of body weight, followed by *in vivo* imaging (Biospace Lab, French). The acquired iMGs were analyzed using M3 Vision software (Biospace Lab, French).

### Statistical Analysis

Statistical analyses were performed using GraphPad Prism 8.0 (GraphPad Software). Data comparisons between two conditions were conducted using unpaired t-tests or one-way ANOVA. All error bars show the Standard Deviation (SD). Statistically significant differences were considered as follows: *P* ≥0.05 (ns), *P* <0.05 (*), *P* <0.01 (**), *P* <0.001 (***) and *P* <0.0001 (****). Fisher's exact test was employed to compare data with specific preferences, with values <0.05 indicate statistical significance.

## Results

### MG represents a unique *piggyBac*-like element

**Figure 1.**
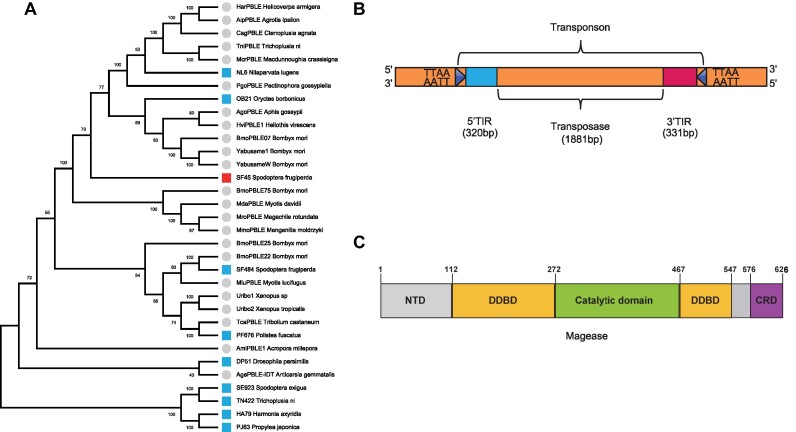
*piggyBac*-like element in Lepidoptera. (**A**) Phylogenetic tree based on the ‘DDE_Tnp_1_7’ domain sequences. (**B**) Diagram of *MG* transposon flanked by TTAA, as well as the arrangement of the 5′ and 3′ TIRs. (**C**) Structure of the *MG* transposase (Magease). N-terminal domain (NTD). Dimerization and DNA-binding domain (DDBD). C-terminal cysteine-rich domain (CRD). Gray indicates disordered regions in the structure.

We identified 33 PLE sequences within the Lepidoptera genome, consisting of 10 consensus sequences obtained through computational analysis (outlined in the Methods section) and an additional 23 sequences sourced from prior publications (66). To understand the evolutionary relationships among these PLEs, we constructed a phylogenetic tree based on the ‘DDE_Tnp_1_7' domain sequences (Figure 1A). Notably, several PLEs (NL6, OB21, SF484, PF676, DP51, SE923, TN422, HA79, PJ63) which, during preliminary tests, displayed no transposase function, hinting at a diminished functional potential. Conversely, the SF45 (hereafter referred to as *Mage* or *MG*), is distinguished by the red square and manifests robust transposase activity in preliminary tests. This indicates that *MG* had the potential to acquire hyperactive activity similar to other PLEs reported in earlier literature. Therefore, our objective was to refine the *MG* system to enhance its transpositional activity.

The *MG* transposase (Magease) is composed of a single open-reading frame (ORF) that codes for a 626-amino acid protein (Figure 1C), with terminal inverted repeats (TIRs) flanking it (Figure 1B). The identification of potential Terminal Inverted Repeats (TIR) upstream and downstream of Open Reading Frame (ORF) sequences involved a series of systematic steps. Candidate regions for TIR were selected by extracting 2 kb upstream and downstream from both the 3′ and 5′ ends of retrieved ORF sequences. Subsequently, 10 bp k-mers were generated from these candidate regions. A comprehensive comparison of all k-mers between the 3′ and 5′ ends ensued, emphasizing k-mers with a mismatch count of less than 3. The positions of matching k-mers were meticulously recorded, facilitating the subsequent grouping of these k-mers based on their positions. Following this, efforts were directed towards expanding these matches to obtain longer segments. fragments exceeding a predetermined length threshold of 12 bp were record as candidate TIR. The candidate TIR sequences then undergo manual verification to confirm their accuracy, ultimately yielding the finalized results for TIR identification. Since the structural domain information for *PB* is known (67), we aligned the*MG* sequence with the *PB* sequence and mapped the structural domain cleavage points of *PB* (i.e. residues 117, 263, 457, 535, 553) to the corresponding alignment positions in *MG*, determining these points to be at residues 112, 272, 467, 547, 576 in *MG*. This information was then used to establish the domain distribution of *MG* (Supplementary Figure S2). The catalytic domain of Magease (spanning residues 272–467), resembling the *PB* transposase structure, adopting the RNaseH-like superfamily fold typical of transposases. Additionally, a unique all-α-helical domain (referred to as the Dimerization and DNA-binding domain, DDBD) forms through the convergence of residues 112–272 and 467–547. This domain plays a crucial role in assembling the protein and interacts with the TIRs. The Cysteine-rich domain (CRD, spanning residues 576–626), linked to the C-terminal end of the DDBD through an extended linker, is indispensable for transposase activity and has been suggested to be responsible for TIR binding.

### The *MG* transposon for efficient transgenesis in diverse mammalian cells.

**Figure 2.**
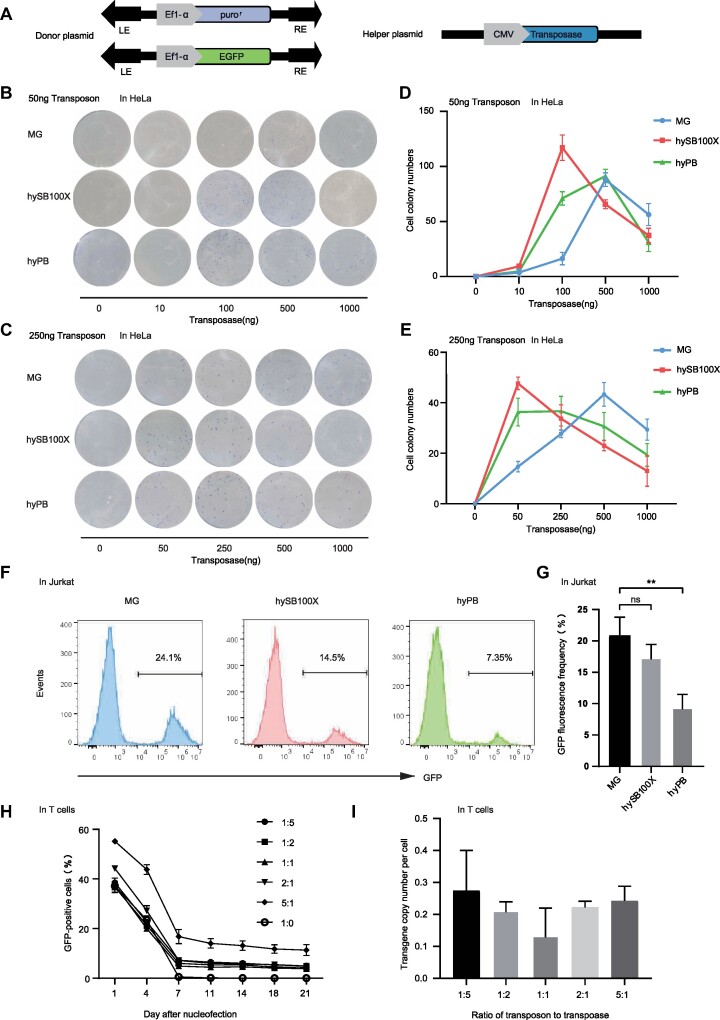
High transposition activity of *MG* in various mammalian cells. (**A**) Donor and helper plasmids are used in human cells. LE and RE indicate the left and right transposon terminal sequences, respectively. EGFP, enhanced green fluorescent protein; Ef1-α, Ef1-α promoter; puro, puromycin resistance gene. Helper plasmids include the CMV promoter and respective coding genes for the transposase (*MG*, hySB100X, hyPB). (**B, C**) Representative images of crystal violet staining in HeLa cell colonies subjected to a high dose of 250 ng transposon and low dose of 50ng transposon. (**D, E**) Comparative analysis of the transposition efficiencies of *MG*, hySB100X and hyPB in HeLa cells, under conditions of 50 ng low-dose and 250 ng high-dose transposon DNA. (**F**) Representative flow cytometry analysis of Jurkat cells electroporated with DNA plasmids carrying the EGFP transposon and transposase. Two weeks after transfection, cells exhibiting stable expression of the integrated EGFP gene were identified. The x-axis represented the intensity of EGFP, and the y-axis represented the frequency of events. (**G**) Graphical representation of GFP-positive T cells 14 days post-electroporation. Data are shown as mean values; error bars, s.d. (*n* = 3 independent experiments). The asterisks denote significant differences, which were determined using Student's t-test. (**H**) Stimulation of primary T cells derived from human peripheral blood mononuclear cells and nucleofection with *MG* at varying doses of EGFP-carrying transposon and transposase plasmids (transposon: transposase ratios of 1:5, 1:2, 1:1, 2:1, 5:1, 1:0). Data are shown as mean values; error bars, s.d. Data were acquired from three independent experiments with one T cell donors. (**I**) Copy number profiles at different ratios of transposon DNA. The copy number determined was normalized to the genomic copy number for RNaseP. Data are shown as mean values; error bars, s.d. Data were acquired from three independent experiments with one T cell donor.

To investigate the transposition efficiency of *MG* within mammalian cells, we compared it with the highly active hyper *Sleeping Beauty* 100X (hySB100X) (39) and hyperactive PBase (hyPB) (41). In this study, we utilized a binary co-transfection assay system (8,68) and incorporated the TIR sequences and transposase expression cassettes of *MG*, hySB100X and hyPB into an identical plasmid backbone to minimize system differences. Initially, we co-transfected HeLa cells with plasmids carrying the puromycin resistance cassette transposon and the transposase plasmid (Figure 2A). To map the impact of varying transposon and transposase plasmid delivery ratios on transposition efficiency and Overproduction Inhibition (OPI)—a phenomenon characterized by a decrease in efficiency as the number of transposases exceeds their optimal range (69)—we co-transfected HeLa cells with transposons at either a high concentration (250 ng) or a low concentration (50 ng), along with transposase concentrations ranging from 0 to 1000 ng. The experimental findings indicate that *MG*, *SB* and *PB* all demonstrate OPI at both high and low transposon concentrations. Specifically, at a lower dose of 50 ng transposon, SB’s peak activity occurs at a transposase concentration of 100 ng, whereas both *MG* and *PB* require 500 ng of transposase to reach their peak activity (Figure 2B, D). In high-dose scenarios, peak activities for *SB* and *PB* are observed at transposase concentrations of 50 ng and 250 ng, respectively. In contrast, *MG* requires a significantly higher transposase concentration of 500 ng to achieve its peak activity (Figure 2C, E, and Supplementary Table S4). Comparative analysis of the peak efficiencies of three transposons reveals that *MG* demonstrates high transposition activity in HeLa cells. Additionally, MG’s cytotoxicity within HeLa cells was analyzed, revealing no significant difference in survival rate between the *MG* and control groups across varied transposon vector dosages (Supplementary Figure S3). Thus, *MG* plasmid overexpression did not correlate with increased cytotoxicity. We subsequently explored whether Jurkat cells could also exhibit active transposition using the *MG* transposon, given Jurkat cells' potential as an economical alternative to T cells in evaluating transposon efficacy. To accurately measure wtMG transposition within Jurkat cells, we employed a fluorescent reporter system (Figure 2A). Following co-transfection with a transposon plasmid bearing the EGFP gene and its respective transposase plasmid, we utilized flow cytometry after two weeks to evaluate transposition efficiency through consistent EGFP reporter gene expression. It is important to note that when using a 1:1 ratio of transposon to transposase, the *MG* exhibits twofold higher activity compared to hyPB (*P* <0.01) and demonstrates comparable transposition activity to hySB100X (*P* = 0.15) (Figure 2F, G).

It is known that the presence of transposon transposition activity in immortalized cell lines does not guarantee efficient and stable gene transfer in primary cells (70,71). Hence, we evaluated the transposition activity of wtMG in primary T cells. Successful MG-mediated transgenesis was accomplished via co-electroporation of T cells with the *MG* transposon harboring the EGFP gene and its transposase, resulting in about 20% stable EGFP expression among transfected cells (Supplementary Figure S4). Interestingly, the integration efficiency of the native *MG* transposon in Jurkat cells mirrored that in T cells. Overall, these findings indicate that MG is a highly active transposon with promise for use as a genetic tool capable of efficiently genetically engineering a variety of mammalian cell types and therapeutically relevant primary human T cells.

### Transposition efficiency of *MG* at different ratios

In many transposon applications, it is necessary to titrate the transposon component to regulate the number of transposon insertions in the genome of each transposon cell. Maintaining a low copy number is advantageous for loss-of-function mutations and therapeutic purposes, while somatic mutations in cancer genes often require a cumulative effect of multiple insertions in the same cells (69). Therefore, we assessed how the *MG*-transfected T cell transposition efficiency varies under different transposon to transposase DNA plasmid ratios, especially given MG’s pronounced transposition activity in T cells. We performed nucleofection of T cells using the transposon donor plasmid pMAX-MG/EF1α-EGFP and the transposase plasmid pCMV-MG at the following ratios: 1:5, 1:2, 1:1, 2:1 and 5:1 (transposon: transposase DNA plasmids). As a control, the transposon cassette alone (1:0) was transfected. To maintain a consistent total amount of electroporated DNA, we supplemented the number of DNA with the control plasmid pCMV-hyPB. The results demonstrated similar integration efficiency for the ratios of 1:5, 1:2, 1:1 and 2:1, with the 5:1 ratio yielding a notably superior integration efficiency (*P*<0.05) (Figure 2H). To assess the correlation between transposable activity and the number of integrations per cell, the transgene copy number of each cell was determined post-electroporation (14 days). The 1:5 and 5:1 ratios manifested analogous copy numbers, as did the 2:1 and 1:2 ratios. Marginally higher copy numbers were observed in the 1:5 and 5:1 ratios compared to the 1:2 and 2:1 ratios, while the 1:1 ratio showed the lowest copy number (excluding the 1:0 ratio data) (Figure 2I).

### Genomic insertion patterns of *MG* in human cells

**Figure 3.**
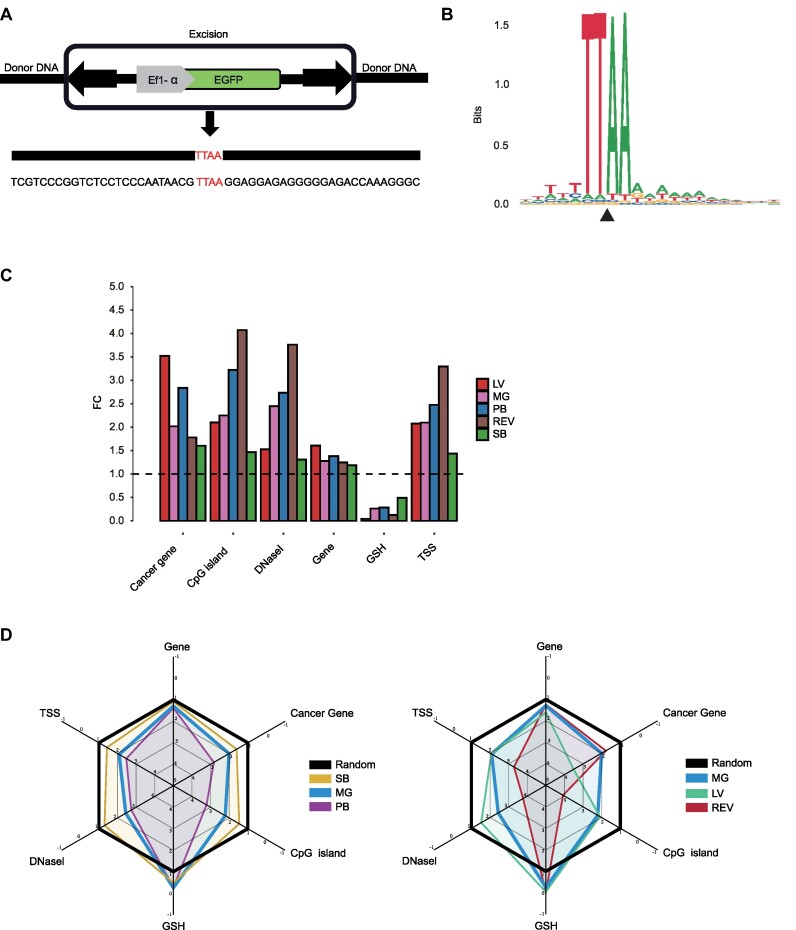
Genome-wide distribution of *MG* transposon insertion sites. (**A**) Footprints resulting from *MG* transposase-mediated excision are highlighted, with the donor TTAA site marked in red. (**B**) The WebLogo diagrams depict common sequences associated with *MG* transposon insertions. The x-axis represents the upstream and downstream regions surrounding the vector integration site. while letter height reflects base conservation. The y-axis indicates a bit of information. The insertion position is indicated by a black triangle. (**C**) Comparative distribution of lentivirus (LV),*Mage* (*MG*), *piggyBac* (*PB*), retrovirus (REV), and *Sleeping Beauty* (*SB*) in the human genome. The random insertion frequency is set to 1, and the vertical coordinate represents the fold change (FC) relative to the random site. ‘GSHs’, genomic safe harbor; ‘TSS’, transcriptional start site. (**D**) Radar charts depicting the distribution of LV, *MG*,*PB*, REV and *SB* in the human genome.

Only the *PB* transposon exhibits a seamless excision property among eukaryotic DNA transposons (9,72). This means that upon removal, the excised genome can restore the original TTAA sequence. In our subsequent excision assay of the *MG* transposon, we made an intriguing discovery: the *MG* transposase demonstrates the same seamless excision property as *PB*. It is capable of excising the *MG* transposon without leaving any trace at the excision site (Figure 3A). To evaluate the integration sites of *MG* at the primary DNA sequence level, we employed sequence logo analysis. The results revealed that the *MG* transposon system integrates into the TTAA target sequence. The central insertion site of TTAA is surrounded by a plural sequence of A or T (Figure 3B), similar to the pattern reported for the *PB* transposon (73).

Recognizing that different transposons exhibit nonrandom target site preferences at the genome-wide scale, we embarked on an exploration of *MG* transposon's insertion bias within the T cell genome. Using high-throughput sequencing, we mapped 4298 integration sites of the *MG* within the genome. In order to compare the integration profiles of *MG* with *SB*, *PB* and viral vector system, we utilized published datasets of human cell integration sites (44,74), and for control, we generated a computational dataset representing random integration. The study results revealed that *MG* exhibited a greater insertion propensity at CpG islands, DNaseI hypersensitivity sites, and transcription start sites (TSSs) compared to lentiviral (LV) and *SB*systems, while displaying a lower insertion propensity in relation to *PB* and retroviral (REV) systems. Specifically, no significant difference was observed between the insertion propensity of *MG* (CpG, FC = 2.25; TSSs, FC = 2.1) and LV (CpG, FC = 2.1; TSSs, FC = 2.08) at the CpG island sites and transcription start sites (*P*= 0.31; *P*= 0.74). Regarding gene regions, *MG* exhibited a slightly higher insertion preference (FC = 1.28) compared to REV (FC = 1.25, *P*= 0.34), higher than *SB* (FC = 1.19, *P*= 1.03 × 10^−9^), but lower than LV (FC = 1.61, *P*= 8.17 × 10^−61^) and *PB* (FC = 1.38, *P*= 0.002). In terms of cancer genes, *MG* exhibited an insertion preference FC of 2.02, which was significantly lower than LV (FC = 3.52, *P*= 1.36 × 10^−7^) and *PB* (FC = 2.84, *P*= 0.04), but slightly higher than REV (FC = 1.78, *P*= 0.54) and higher than *SB* (FC = 1.6, *P*= 0.005). When evaluating insertion tendencies within genomic safe harbors (GSH), *SB* exhibited the highest propensity, followed by *PB* and *MG* (Figure 3C). Collectively, compared to *PB*, LV and REV, *MG* displays a weaker insertion tendency at some sites, leaning more towards the insertion pattern of random (Figure 3D).

### Artificial intelligence (AI)-assisted optimization of *MG*

**Figure 4.**
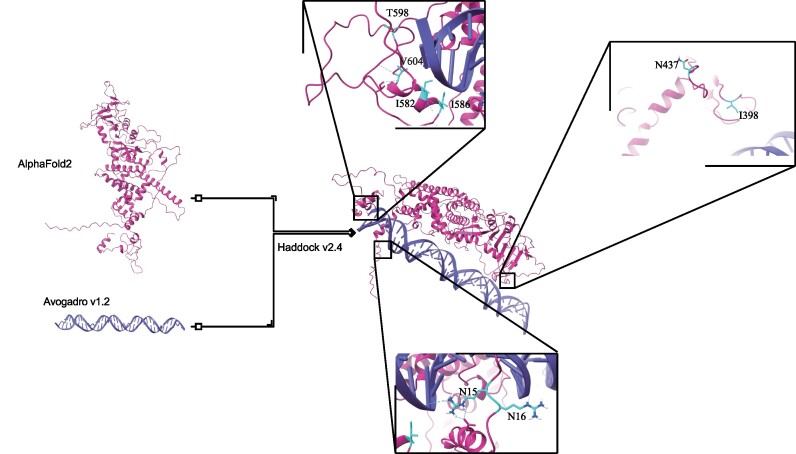
Diagram of artificial intelligence (AI)-assisted optimization of *MG* transposase protein. Take note of the two distinct amino acid mutations at position N437 (N437T and N437E).

Historically, the original *SB* and *PB* have undergone extensive optimizations to enhance transposition efficiency using a plethora of protein engineering techniques post-discovery (41,75). In a parallel vein, our study aimed to augment the wild-type *MG* (wtMG). Rather than haphazardly producing *MG* mutations, we adopted a systematic approach by combining plausible mutations in structural models with experimental mutagenesis tests. Initially, we generated a structural model of the wtMG transposase protein (Magease) using the state-of-the-art AlphaFold v2.0 (59) algorithm, which employs a deep neural network for end-to-end modeling. The prediction results from AlphaFold indicate that the model has a good level of confidence (pLDDT >80) for most residues, which suggests that the backbone is expected to be modeled well (Supplementary Figure S5). Concurrently, we crafted a model of TIR DNA utilizing the Avogadro v1.2 (60) software, which structures the predominant B-DNA conformation found in cells. Armed with these optimally predicted *in silico* models, we harnessed Haddock v2.4 (61) to construct protein-DNA complexes (Figure 4). These final docking outcomes were hierarchically arranged based on the HADDOCK (61) score (details in Methods).

**Figure 5.**
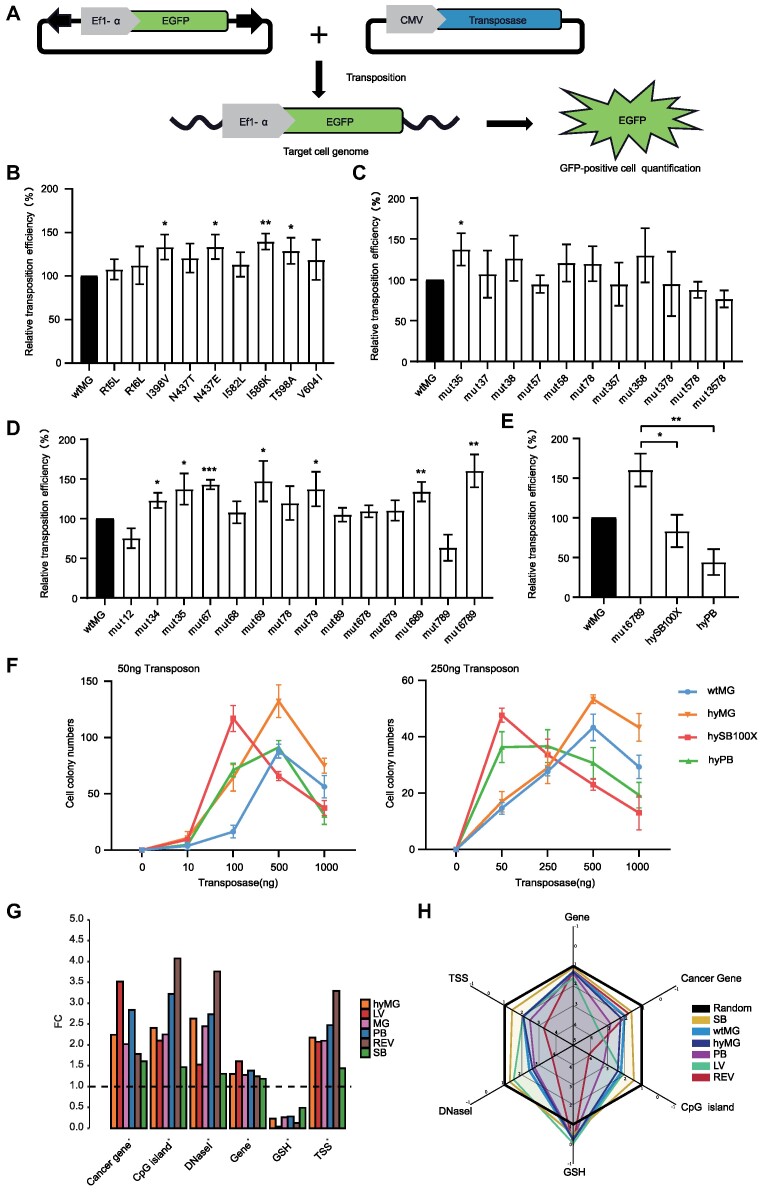
Transposition activities of *MG* transposase mutants. (**A**) A schematic overview of transposition. (**B**) The relative transposition frequency of *MG* transposase mutants was determined by analyzing GFP fluorescence expression through FACS analysis and normalized to wtMG. wtMG, the positive control, was set at 100%. Data are shown as mean values; error bars, s.d. (*n* = 3 independent experiments). (**C**) Highly active single amino acid switch combinations. Data are shown as mean values; error bars, s.d. (*n* = 3 independent experiments). (**D**) Same region combination mutation. Mutant 1, R15L; 2, R16L; 3, I398V; 4, N437T; 5, N437E; 6, I582L; 7, I586K; 8, T598A; 9, V604I. Data are shown as mean values; error bars, s.d. (n = 3 independent experiments). (**E**) Comparison of the relative efficiencies of the wtMG, the mutant 6789, hyPB, and hySB100X. Data are shown as mean values; error bars, s.d. (*n* = 3 independent experiments). All asterisks denote significant differences, which were determined using Student's t-test. (**F**) Comparative analysis of the transposition efficiencies of wtMG, hyMG, hySB100X and hyPB in HeLa cells, under conditions of 50 ng low-dose and 250 ng high-dose transposon DNA. (**G**) Comparative distribution of LV, wtMG, hyMG, *PB*, REV and *SB* in the human genome. The random insertion frequency is set to 1, and the vertical coordinate represents the fold change (FC) relative to the random site. (**H**) Radar charts depicting the distribution of LV, wtMG, hyMG, *PB*, REV and *SB* in the human genome.

With the AI-assisted approach, we prioritized 39 *MG* AAs which were shown *in silico* as highly relevant in Magease binding to its target DNA molecules. To derive the hyperactive version of the *MG* transposase, we undertook transposase engineering by single-amino-acid substitutions based on the Magease-DNA complexes and the putative 39 mutagenesis points described in Supplementary Table S5. Subsequently, we evaluated the transposition efficiency of these 39 mutant transposases within Jurkat cells. During this assay, a donor plasmid carrying the EGFP expression gene transposon was co-transfected with a helper plasmid encoding either the wtMG transposase or a mutant variant (Figure 5A). Out of the 39 predicted mutant transposases, we identified nine candidates that exhibited higher relative transposition efficiency relative to the wtMG (Figures 4 and 5B, and Table 1). Four mutations, specifically I398V (133%, *P*= 0.015), N437E (134%, *P*= 0.014), I586K (140%, *P*= 0.002), and T598A (129%, *P*= 0.029), were particularly prominent in their efficiency (Figure 5B). Further analysis of the domain organization of the MG transposase revealed specific amino acid substitutions associated with these mutations: R15L and R16L in the N-terminal domain; I398V, N437T and N437E in the catalytic domain; and I582L, I586K, T598A and V604I in the C-terminal cysteine-rich domain (CRD) (Table 1).

**Table 1.** List of individual amino acid residues of Magease

NO.	Amino acid residue in Magease	Domain	Change made
Mutation 1	R15	N-terminal	L
Mutation 2	R16	N-terminal	L
Mutation 3	I398	Catalytic domain	V
Mutation 4	N437	Catalytic domain	T
Mutation 5	N437	Catalytic domain	E
Mutation 6	I582	CRD	L
Mutation 7	I586	CRD	K
Mutation 8	T598	CRD	A
Mutation 9	V604	CRD	I

Our next endeavor was to determine if combining these mutant sites could synergistically boost transposition efficiency. Two methods of mutation combination were pursued: (i) integrating the four most efficient single amino acid mutations and (ii) combinations of mutations within the same domain. The first strategy involved combining four highly active mutants (I398V, N437E, I586K and T598A). Overall, most of the mutant combinations displayed substantial levels of transposition activity compared to wtMG, indicating the viability of this combination approach. Notably, mutation 35 (*P* = 0.031) exhibited the highest transposition efficiency at 137% relative to wtMG (Figure 5C). Conversely, a group of mutants represented by 57, 357, 378, 578 and 3578 showed a moderate reduction in transposition efficiency, ranging from 76% to 95% compared to wtMG (Figure 5C). Our second approach was domain-specific to minimize interference between mutations. Specifically, we considered two mutations in the N-terminus region (R15L and R16L), three mutations in the catalytic domain (I398V, N437T and N437E), and four mutations in the CRD (I582L, I586K, T598A and V604I) as combinations within the same domain (Figure 5D). Here, mutation combinations 6789 (160%, *P*= 0.007), 69 (147%, *P*= 0.007) and 67 (143%, *P*= 0.0002) excelled in their performance. However, combinations 12 and 789 were less effective than the wtMG (Figure 5D). Certain combinations indeed manifested synergistic effects, with mutation 6789 (I582L, I586K, T598A and V604I) emerging as the top performer. Remarkably, this mutation, now termed hyMage (hyMG), outperformed renowned transposons like hyPB and hySB100X in Jurkat cells under a 1:1 transposon-to-transposase ratio, outstripping their efficiency by approximately 2-fold and 3-fold respectively (Figure 5E). Subsequent experiments evaluated hyMG’s transposition efficiency in HeLa cells at both high (250 ng) and low (50 ng) transposon concentrations (Same batch of experiments as Figure 2D, E). These tests revealed that hyMG requires 500 ng of transposase to achieve peak activity in both scenarios, and in each case, it exhibited OPI, consistent with the data for wtMG. Notably, hyMG’s peak activity surpassed that of wtMG, hySB100X, and hyPB at both low and high transposon concentrations (Figure 5F). Further investigations confirmed that hyMG primarily integrates into the TTAA locus (Supplementary Figure S6). Utilizing high-throughput sequencing, we effectively mapped 7996 integration sites of hyMG within the genome, underscoring that hyMG's insertion preferences closely align with those of wtMG (Figure 5G, H).

### Effects of NKG2D CAR T cell generation by hyMG

**Figure 6.**
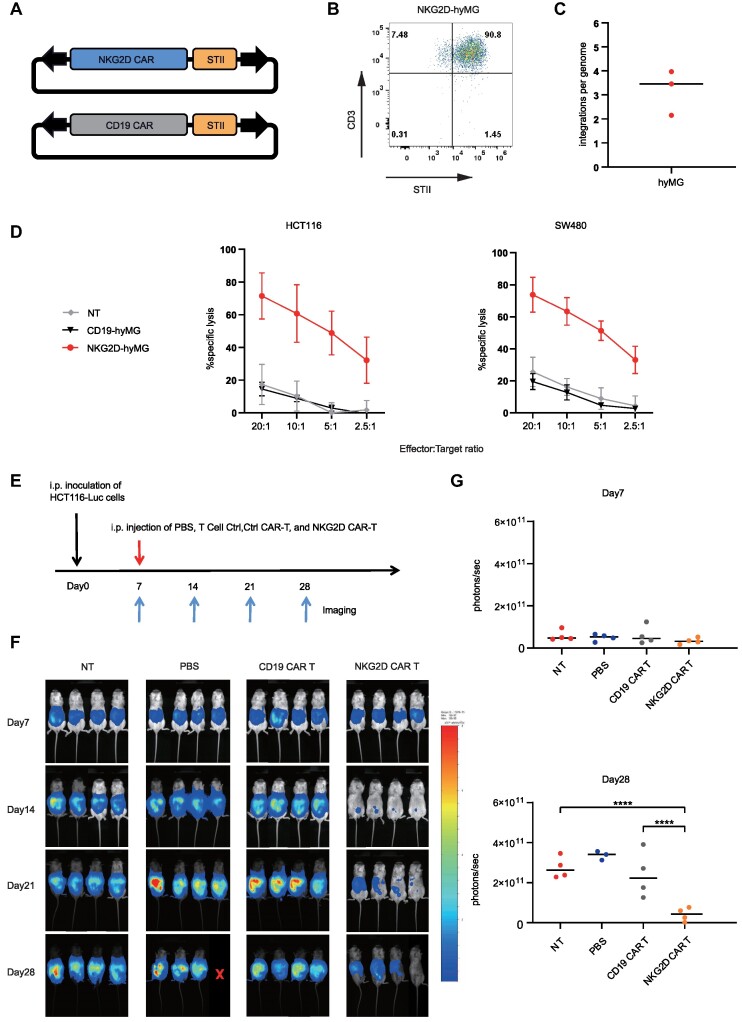
hyMG-generated NKG2D CAR T cell and functional analysis. (**A**) NKG2D CAR and control CAR constructs were utilized in this study. To facilitate the detection of CAR expression through flow cytometry analysis, a Strep-Tag II (STII) tag was incorporated. (**B**) Representative flow cytometry analysis of T cells transfected with NKG2D CAR hyMG transposons. T cells were obtained from healthy donors and enriched for CAR-positive cells through co-culture with K562A. The x-axis represents fluorescence from STII, and the y-axis represents fluorescence from CD3. The experiment was repeated three times, each time using cells from three different T-cell donors. (**C**) The average number of NKG2D CAR transgene insertions in the genome of CAR T cells was determined through three independent assays using cells from three different T-cell donors. Data are shown as mean values; error bars, s.d. (**D**) Cytolytic activity of NKG2D CAR T cells generated using hyMG transposons. The Delfia EuTDA cytotoxicity assay (4-hour EuTDA culture) was employed to evaluate tumor cell lysis efficiency. Data were acquired from three independent experiments with three different T cell donors. Data are shown as mean values; error bars, s.d. (**E**) Experimental procedure for animal studies using hyMG-generated NKG2D CAR-T cells. NCG mice were i.p. injected with 2 × 10^6^ HCT116-luc cells on day 0. Four groups of mice (4 mice per group) received i.p. injection of PBS, T cells (NT), ctrl T cells (CD19 CAR-T), and NKG2D CAR-T cells on day 7 (1 × 10^7^ cells per mouse). On days 7, 14, 21 and 28, bioluminescent imaging of tumor signals was carried out. (**F**) The bioluminescence image for the indicated time is displayed. (**G**) The bioluminescence flux measurements from each mouse in the corresponding group were plotted on days 7 and 28. Asterisks denote significant differences, which were determined using Student's t-test.

To investigate the therapeutic potential of *MG* in primary human cells, we focused on a CAR T system developed using *MG* plasmid electroporation. The chosen CAR targeted NKG2D, a natural killer (NK) cell receptor known for its efficacy in cancer treatment based on preclinical animal models (76,77). This NKG2D CAR plasmid was designed to include the NKG2D ectodomain, the 4–1BB (CD137) costimulatory domain, and the DAP12 activation domain, with Strep-Tag II added as a CAR verification tag (STII) (Figure 6A, Supplementary Figure S7). Following CAR creation, NKG2D CAR T cells were cultivated through the stimulation of human peripheral blood mononuclear cells (PBMCs) and electroporation with the CAR-encoding *MG* transposon. Post co-culturing with gamma-irradiated K562A cells, which naturally exhibit NKG2D ligands, we observed a significant uptick in NKG2D CAR positivity, at 87.1% ± 3% by day 11 (Figure 6B, Supplementary Figure S8). Subsequently, genomic DNA from the enriched NKG2D CAR T cells was analyzed to determine the copy number of CAR transgenes, which was found to be an average of 3.2 ± 0.8 copies per cell (Figure 6C). Functional assessment of these CAR T cells revealed potent lysis capabilities against targeted cancer cell lines, a stark contrast to the performance of control cells (Figure 6D). *In vivo* trials, conducted using a mouse model (NOD scid gamma mice with HCT116 cell injections), divided mice into groups receiving varying treatments: PBS, control T cells, CD19 CAR T cells, and NKG2D CAR T cells (Figure 6E). Remarkably, the hyMG-generated NKG2D CAR T cells induced rapid tumor regression within a week after CAR T cell transfer and maintained control over tumor growth for 28 days (Figure 6F, G). This underscores hyMG's potency in stably integrating the NKG2D CAR into T cells, offering promising therapeutic outcomes in mice.

## Discussion

Through bioinformatic exploration, we identified an innovative *piggyBac*-like element termed *MG*. Exhibiting robust efficacy across a spectrum of mammalian cells, *MG* retains the seamless excision attributes seen with *PB*. This attribute, in conjunction with its ability to consistently generate CAR T cells, underscores its potential for cancer treatment.

In specific cellular environments, including HeLa, Jurkat, and T cells, the native *MG* displayed pronounced activity (Figure 2). Our investigation extended to OPI of the transposon—a crucial biological characteristic. OPI may be involved in transpososome construction due to the multimeric state of transposases and the competition for binding sites inside transposon TIRs, according to some theories (78). Experimental findings revealed that *MG* transposons exhibited OPI at both low (50 ng) and high (250 ng) transposon concentrations in HeLa cells. In comparison, OPI of *SB* was slightly more sensitive and reached peak activity at lower transposase concentrations whereas the *MG* did not reach peak activity until the transposase concentration reached 500 ng (Figure 2). OPI demonstrated by *SB* is extensively documented in the literature (13,14,69,79,80). Our *in vitro* assessments, spanning various transposon-to-transposase plasmid ratios, spotlighted 5:1 as the most effective ratio. Notably, other ratios did not show significant differences in gene transfer efficiency (Figure 2). Furthermore, the number of integrations in cells does not correlate directly with transposable activity. The integration count is determined by the cumulative amount of electroporated plasmids, with higher numbers resulting in elevated cellular copy numbers (Figure 2). Hence, by modulating the quantity and ratio of transposons to transposases, we can control genomic transposon copy numbers, thereby reducing potential hazards associated with *MG* transposons causing host genome mutations.

Integration site preference of transposons is a critical biological characteristic that profoundly impacts the suitability of transposon vectors for various applications (36). For instance, a non-preferential transposon system is ideal for human gene therapy, while those favoring gene and gene regulatory region integration might be better suited for mutagenesis applications. To assess potential insertional mutations for gene therapy, we investigated the integration sites of the *MG* element in primary T cells. We employed tagmentation-assisted PCR to efficiently recover a large number of mammalian insertion sites, subsequently analyzed via next-generation sequencing (63). This approach provides a comprehensive and precise approach for investigating transgenic insertion sites and offers the advantages of rapid and straightforward sample preparation, surpassing restriction endonuclease-based techniques. Our in-depth analysis illuminated MG's inclination for integration proximate to genes, transcriptional start sites, CpG islands and DNaseI sites. Interestingly, compared to *PB*, MG’s proclivity towards these areas was somewhat reduced (Figure 3). Additionally, we observed that *MG* exclusively integrates TTAA tetranucleotides, similar to *PB* (72) (Figure 3). These findings enhance our understanding of MG’s potential as an enhancer-trapping genetic tool and improve the safety profile of *MG* in gene therapy. Notably, excision assay have shown that *MG* shares the same seamless excision properties as *PB* (41) (Figure 3). This means that *MG* is capable of precisely repairing gaps caused by excised transposons without the need for DNA synthesis, which is a highly attractive advantage. In contrast, transposons that require DNA synthesis for gap repair may lead to undesired mutations in host genes if their excision generates footprints. The presence of active transposase in the cell allows transposons, including *MG* and *PB*, to be recognized and reintegrated into new positions in the genome. The seamless excision properties of *MG* and *PB* transposons ensure that they do not introduce unwanted footprints when transposase persistence is maintained.

The reverse repeat sequence at the end of the transposon and the transposase protein are both essential for transposons. Manipulating these elements can enhance transposon activity. In this study, we improved the transposition efficiency of *MG* through *in silico*virtual screening and feasible combinatorial mutagenesis in vitro. Among the 39 potential mutations examined, 9 individual amino acid alterations (23%) markedly elevated *MG* transposase efficacy. While specific mutation combinations exhibited synergistic enhancements, others did not, likely due to structural rearrangements induced by amino acid changes that impeded transposition efficiency. Ultimately, we identified the most effective mutation combination, referred to as hyMG (Figure 5). Within Jurkat cells, hyMG demonstrated superior performance compared to both hyPB and hySB100X when subjected to a 1:1 transposon-to-transposase ratio. Similarly, in HeLa cells, the peak activity of hyMG surpassed that of hySB100X and hyPB, as evidenced in experiments utilizing varied transposon and transposase ratios (Figure 5). These findings expand the potential applications of transposons as powerful genome engineering tools. Nevertheless, the precise mechanisms underpinning each mutation's influence on transposition frequency remain enigmatic and warrant further exploration. Overall, the results showed that compared to traditional mutagenesis methodologies (41,75), the AI-assisted virtual screening approach is a substantial leap forward in transposase engineering and structural understanding when juxtaposed with traditional mutagenesis strategies.

DNA transposons have emerged as promising non-viral vectors for immunotherapeutic applications, particularly in gene therapy applications of CAR T. In our study, we successfully constructed a CAR T system with hyMG and observed that a single infusion of NKG2D CAR T cells for tumor treatment in HCT116 intestinal cancer-bearing mice and inhibited tumor progression after 28 days (Figure 6). It is conceivable that multiple CAR T cell infusions could enhance therapeutic outcomes. Given the inherent risk of insertional tumorigenesis associated with all transposon vectors, and this risk escalates with the number of copies of the inserted transgene, regulatory agencies in the US and Europe mandate a limit of fewer than five copies per genome (81). In our NKG2D CAR-modified T cells, the hyMG transposon generates approximately three copies of the inserted gene (Figure 6). Alternatively, we can mitigate safety concerns by minimizing the number of inserted copies through the reduction of transposons or transposase input.

The discovery of *MG* has brought forth exciting possibilities in the realm of cell and gene therapy. Our study has demonstrated the robust transposition capability of MG in various mammalian cells and primary T cells, comparable to the highly optimized *SB* and *PB* systems. Nevertheless, there is still untapped potential for further optimizing MG. For instance, structural modeling techniques were employed to generate engineered variants of the SB transposase, strategically inserting them away from gene regulatory regions and exons, thus elevating their safety for gene therapy applications (82). Additionally, a highly soluble *SB* (hsSB) was obtained by mutagenesis of SB transposase, which enables efficient genetic modification in induced pluripotent stem cells (iPSCs) without the need for transfection reagents, thereby offering a promising avenue for therapeutic genome engineering (40). For *SB* to be truly translated into clinical practice, a comprehensive investigation of its safety profile is imperative (83,84). Given the immense therapeutic potential of *MG*, future research demands rigorous safety validation studies to ensure its successful clinical application.

Overall, this study presents a potent non-viral transgenic tool that exhibits high activity in both diverse mammalian cell lines and primary T cells. This tool enables the genetic modification of T cells to express specific chimeric antigen receptors (CARs) and achieve gene therapy effects, thereby creating a new avenue for gene manipulation.

## Supplementary Material

gkae048_Supplemental_File

## Data Availability

The PLE sequences can be found at the NCBI with following accession. NL6: AOSB01030598.1: 24655-27546, OB21: CACRWG010000127.1: 313136-315929, SF484: OEOA01000549.1: 7106-11436, PF676: QUOH01000056.1: 13385-16023, DP51: AAIZ01010985.1: 6921-9758, SE923: WNNL01000012.1: 9873898-9876378, TN422: NKQN01000830.1: 4214-6714, HA79: WMPA01000160.1: 1315089-1317567, PJ63: JACDNR010001066.1: 104072314-104074866. The sequencing data for this study have been deposited in the European Nucleotide Archive (ENA) at EMBL-EBI under accession number PRJEB66209. The *MG* transposase protein and TIR DNA complexes structural model is available in ModelArchive at https://www.modelarchive.org/doi/10.5452/ma-zxdgf.
