# Supplementary material for: Mage transposon: a novel gene delivery system for mammalian cells
Source: Nucleic Acids Res. 2024 Feb 1;52(5):2724–39. doi: 10.1093/nar/gkae048 (PMC10954464; doi:10.1093/nar/gkae048)
Supplement: gkae048_Supplemental_File [file gkae048_supplemental_file.pdf]

SUPPLEMENTARY FIGURES AND LEGENDS

Supplementary Table S1. Sequences of MG TIRs and transposase.

Name	Sequence (5’-3’)
MG 5’TIR	ttaacccttatgtgagtgacaaataaaacacctacatcagagtgacaaggtacccgggtaccttcat<br>acagtttatacgtaatgcaaacaatcaagtactgttttaacgttacccacacttttaattttattactttagtt<br>aagataaacaattgacggtctgtggggattaaagggggggagcggtggagtgcggtacatggttatt<br>ggcggtagaaacactgagtttattcaatgcatcgtgcttgacattgaaacttgtctgattactttttttcgtgt<br>tgtagaagcctacttctattttgtgagtttataattatttcatt
MG 3’TIR	acaacaaaattgtattttttatagaaataaaacattgtaacaactttttattatttcatttttgtccaaaattat<br>aaatactttttttcattttcatacaagacacaaagaggtacccgggtacctagtcactctattgtgagttca<br>aaatatgttttggtttccacaaaacatagcaaacgattgactgggtcaaaacattaaaaaatatgtaa<br>tttataaatagttaggagctctcaggaaatcacagctacctatagagtgtaaaacgtacttttctttaaat<br>ttgaaattgctaaaggtacccgggtacctcgtcacacacataagggttaa

MG transposase (codon optimized)	atggatatcggagcctctacaagtaaacagaggtcacgcatccggcggtataatagtacggaaatt<br>caagctattctggcggagagttcttcagatgaaggcgacttcgtggcatccgacaatgaacacgact<br>acatacctctcgtagaggaaggcggtctcgagatcagcgatgtagagccagaggcggaagttga<br>aaatgaaaacgactcctacgacagtgacgcgagcttcgagcgggaatcttttgaatcaattcttaca<br>gcaaaggatggaacgaggtggcaatcagagccattgccgcgcacacagacctcaagccgaaa<br>cattatacgccaacggggcggccctgcttccttttgtaagctttattctcaaatggaagtcttcaagagt<br>atactctcaaacgacatgtgtgacattatactcagagagacaaataggaagggaacaaagatcac<br>tgaagcttataacaataagctgatggaaaattaccccgatgtctcaaagcgacccaaacagaaaa<br>tcttcaaacttttcacagagcaagaactggacgcattctttggaatccttattttcagtggagtgcatcga<br>agtaataaggaacatctcaccgagctgtggaaatccaaccacttgccgctgttccgagcagccatg<br>tcccacgacagattcaaaatgctgctgcgatttataagatttgataacgatgtcacgcggccagaga<br>ggcttctggctgataaagcgacggctatacgagacatctggaccatgttcattagcaatcttaataaa<br>atgtacaaaccgcgggaatgcataactgtagacgagcagctttatggttataggggacgcactaga<br>tttactcagtacatgccctctaagccagaaaaatacggtatcaagatattctgggcgtgcgacgctaa<br>caattcatatccattgaacgggcagatatacaccgggaagagttccgatggaaacagacagaag<br>aatgtcggcgaatgtacggtactggatttggtagccaagtacaaaaactctggtcttaatgtgactac<br>cgataactttttacctcactccaactggctcatagtctcaatagttggaatatgaccctggttggaactg<br>tacggaaaaacaaacggttcctgcctgccaacatgcaggcccacaaagagcgggtaatttattcat<br>ccaactttgcattttccaaggaagctactgtctgttcatacgttcctaagaagaataaggctgtaatcat<br>gctcagttctatgcatatgtcaccagttatcgagtccaataaggaaacggcgaagccagacataatt<br>ctgtattacaacaaaactaaaagtggcgtcgataacatggataaactcctggcagaatataccgtc<br>aagaggcgcaccaacagatggcccctcgcgctctttttaatattatagatatagccgcgctggcgg<br>cttatatcatatatatggagcataaccctcagtttgtaagctccgataggcggcgcaagttcctgaaat<br>ctctgtccttgcagctttgcgcacagaacatcgaagaaagatcaaagaactgtattgtcacctccaa<br>acttcatgtccgatcagcaatgcaggatgtgctgggacaagagctgcgccttcctataccctctggg<br>agcgctactttgaccaaccagactcgacgggactccactggcagagtagctgtggtcggatcttgtt<br>atatatgccgagagatcaaacgaaagcagcggaagactcgaaaggcctgcactaattgcagga<br>aacccgtgtgtgatgaacatgccgttacgcaccccatctgcaacacatgcttcgaaagttgtgccaa<br>cagtaagtaa

Supplemental Table S2. Table of primers used for Tag-PCR amplification.

Round	Primer name	Sequence (5'-3')
1st PCR	NTSR1-F1	TCGTCGGCAGCGTCAGAT
	NTSR2-F1	GTCTCGTGGGCTCGGAGATG
	Mage 5'IR-R	TGTGGGTAACGTTAAAACAGTACTTGATTGTTTGC
	Mage 3'IR-F	TAGCAAACGATTGACTGGGTCAAAACATTAAAA
2nd PCR	Nextera-2nd-i5-N501-5'IR	AATGATACGGCGACCACCGAGATCTACACTAGATCGCTCGTCGGCAGCGTCAGATGTGTATAAGAGACAGCCGGGTACCTTGTCACTCTG
	Nextera-2nd-i5-N501-3'IR	AATGATACGGCGACCACCGAGATCTACACTAGATCGCTCGTCGGCAGCGTCAGATGTGTATAAGAGACAGGAGCTCTCAGGAAATCACAGC
	Nextera-2nd-i5-N502-5'IR	AATGATACGGCGACCACCGAGATCTACACCTCTCTATTCGTCGGCAGCGTCAGATGTGTATAAGAGACAGCCGGGTACCTTGTCACTCTG
	Nextera-2nd-i5-N502-3'IR	AATGATACGGCGACCACCGAGATCTACACCTCTCTATTCGTCGGCAGCGTCAGATGTGTATAAGAGACAGGAGCTCTCAGGAAATCACAGC
	Nextera-2nd-i5-N503-5'IR	AATGATACGGCGACCACCGAGATCTACACTATCCTCTTCGTCGGCAGCGTCAGATGTGTATAAGAGACAGCCGGGTACCTTGTCACTCTG
	Nextera-2nd-i5-N503-3'IR	AATGATACGGCGACCACCGAGATCTACACTATCCTCTTCGTCGGCAGCGTCAGATGTGTATAAGAGACAGGAGCTCTCAGGAAATCACAGC
	Nextera-2nd-i5-N501	AATGATACGGCGACCACCGAGATCTACACTAGATCGCTCGTCGGCAGCGTC
	Nextera-2nd-i5-N502	AATGATACGGCGACCACCGAGATCTACACCTCTCTATTCGTCGGCAGCGTC
	Nextera-2nd-i5-N503	AATGATACGGCGACCACCGAGATCTACACTATCCTCTTCGTCGGCAGCGTC
	Nextera-2nd-i7-N723-5'IR	CAAGCAGAAGACGGCATACGAGATGAGCGCTAGTCTCGTGGGCTCGGAGATGTGTATAAGAGACAGCCGGGTACCTTGTCACTCTG
	Nextera-2nd-i7-N723-3'IR	CAAGCAGAAGACGGCATACGAGATGAGCGCTAGTCTCGTGGGCTCGGAGATGTGTATAAGAGACAGGAGCTCTCAGGAAATCACAGC
	Nextera-2nd-i7-N724-5'IR	CAAGCAGAAGACGGCATACGAGATCGCTCAGTGTCTCGTGGGCTCGGAGATGTGTATAAGAGACAGCCGGGTACCTTGTCACTCTG
	Nextera-2nd-i7-N724-3'IR	CAAGCAGAAGACGGCATACGAGATCGCTCAGTGTCTCGTGGGCTCGGAGATGTGTATAAGAGACAGGAGCTCTCAGGAAATCACAGC
	Nextera-2nd-i7-N727-5'IR	CAAGCAGAAGACGGCATACGAGATACTGATCGGTCTCGTGGGCTCGGAGATGTGTATAAGAGAC

	AGCCGGGTACCTTGTCACTCTG
Nextera-2nd-i7-N727-3'IR	CAAGCAGAAGACGGCATACGAGATACTGATCG GTCTCGTGGGCTCGGAGATGTGTATAAGAGAC AGGAGCTCTCAGGAAATCACAGC
Nextera-2nd-i7-N723	CAAGCAGAAGACGGCATACGAGATGAGCGCT AGTCTCGTGGGCTCGG
Nextera-2nd-i7-N724	CAAGCAGAAGACGGCATACGAGATCGCTCAGT GTCTCGTGGGCTCGG
Nextera-2nd-i7-N727	CAAGCAGAAGACGGCATACGAGATACTGATCG GTCTCGTGGGCTCGG

*Blue and purple letters indicate incomplete adapter sequences for P5 and P7 adapters, respectively. Green letters indicate sequences for completing adapters

Supplementary Table S3. Pairing of primers for 1st PCR and 2nd PCR.

Round	Primer1	Primer2
1st PCR	NTSR1-F1	Mage 5’IR-R
	NTSR1-F1	Mage 3’IR-F
	NTSR2-F1	Mage 5’IR-R
	NTSR2-F1	Mage 3’IR-F
2nd PCR	Nextera-2nd-i5-N501	Nextera-2nd-i7-N723-5’IR
	Nextera-2nd-i5-N502	Nextera-2nd-i7-N724-5’IR
	Nextera-2nd-i5-N503	Nextera-2nd-i7-N727-5’IR
	Nextera-2nd-i5-N501	Nextera-2nd-i7-N723-3’IR
	Nextera-2nd-i5-N502	Nextera-2nd-i7-N724-3’IR
	Nextera-2nd-i5-N503	Nextera-2nd-i7-N727-3’IR
	Nextera-2nd-i7-N723	Nextera-2nd-i5-N501-5’IR
	Nextera-2nd-i7-N724	Nextera-2nd-i5-N502-5’IR
	Nextera-2nd-i7-N727	Nextera-2nd-i5-N503-5’IR
	Nextera-2nd-i7-N723	Nextera-2nd-i7-N723-3’IR
	Nextera-2nd-i7-N724	Nextera-2nd-i7-N724-3’IR
	Nextera-2nd-i7-N727	Nextera-2nd-i7-N727-3’IR

Supplemental Table S4. Colonies of HeLa cells transfected with wtMG, hyMG, hySB100X and hyPB transposases (0-1000 ng) were counted at different transposon DNA concentrations (50 and 250 ng).

Tn:Tpase (ng)	wtMG			hyMG			hySB100X			hyPB		
Low dose												
50:0	0	0	0	0	0	0	0	0	0	0	0	0
50:10	5	6	0	13	4	15	10	7	11	6	5	3
50:100	10	21	18	57	58	78	129	116	106	64	74	75
50:500	90	93	81	122	126	149	65	62	70	86	90	98
50:1000	45	60	64	76	81	68	45	33	34	40	29	24
High dose												
250:0	1	0	0	0	0	0	0	0	0	0	0	0
250:50	13	17	14	20	18	13	45	50	48	30	40	39
250:250	26	28	29	34	23	32	30	31	40	41	30	39
250:500	47	38	45	55	53	52	25	21	23	28	27	37
250:1000	34	26	28	40	41	49	20	10	9	15	24	19

Supplementary Table S5. Mutation residues.

position	amino acid	basic mutation	acidic mutation	hydrophile mutation	ohter mutation	domain
10	Q		E			C-terminal
15	R				L	
16	R				L	
17	Y		E			
198	H	K			M	DDBD
199	R	K				
200	S		D			
202	K	H				
211	S		D			
213	H			S		
218	R			S		
397	V		E	T		Catalytic domain
398	I				V	
399	Y				G	
417	K	R				
418	K				P	
437	N		E	T		
440	T	K				
461	K		E		Q	
581	Y		E	T	Q	CRD
582	I		E		L	
586	I	K				
598	T	K	E		A	
599	N	K				
602	K	R				
604	V				I	
609	A			S	N	
612	H	K			I	

Figure S1

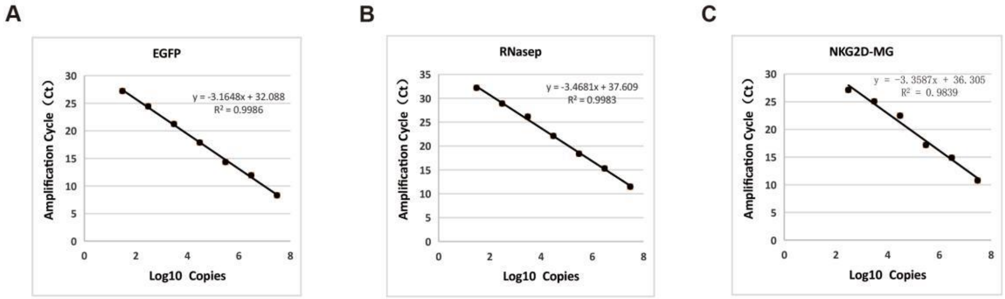

Supplementary Figures S1. Quantitative PCR standard curves.

Standard curves of Q-PCR for EGFP(A), RNase P(B), and NKG2D-MG(C). For linear regression, mean Ct values from qPCR were utilized, with each experiment conducted in triplicate.

Figure S2

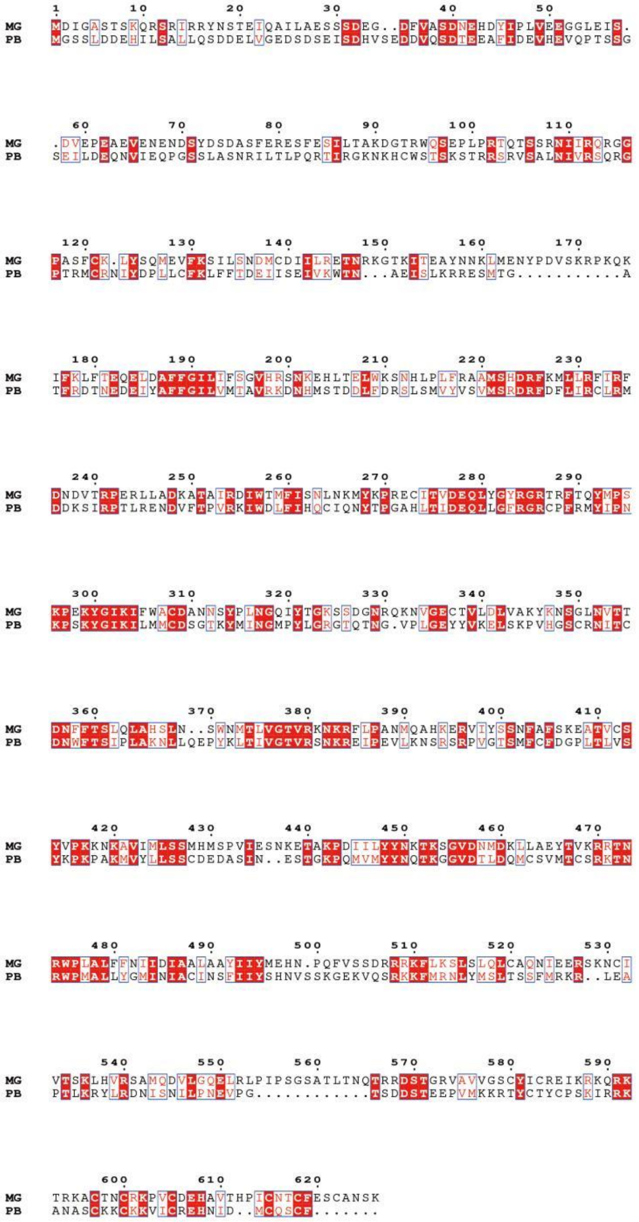

Supplementary Figures S2. Alignment of the transposase of MG and PB.

The structural domains of the MG transposase were identified based on hmmscan predictions and sequence alignment of MG with PB.

Figure S3

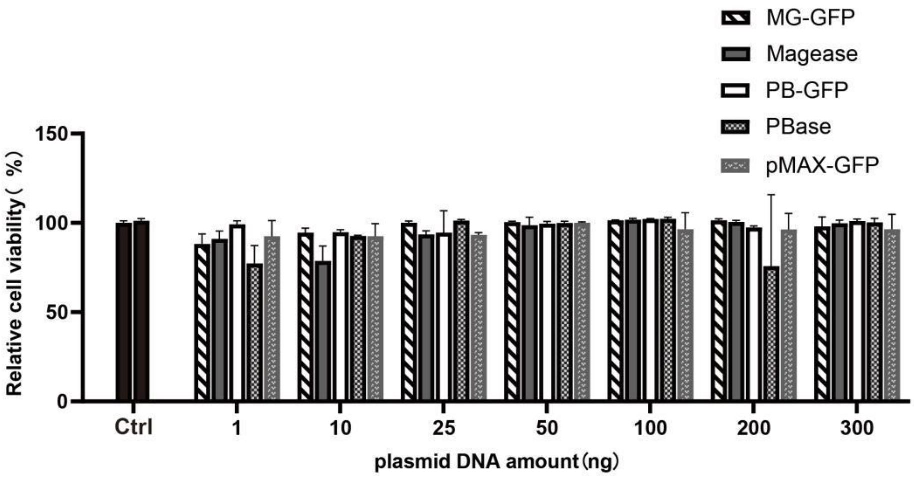

Supplementary Figures S3. HeLa cell viability with MG plasmid expression.

HeLa cells were transfected with different quantities of transposon vectors to assess the potential cytotoxicity associated with MG transposon plasmid expression. The control groups consisted of HeLa cells and those transfected with lipo3000 transfection reagent, denoted as Ctrl. Cell viability was assessed using the CCK8 assay. No statistically significant difference was observed between the MG transposon and the control groups (one-way ANOVA). Data are presented as mean values with standard deviations (SD) (n = 3 independent experiments).

Figure S4

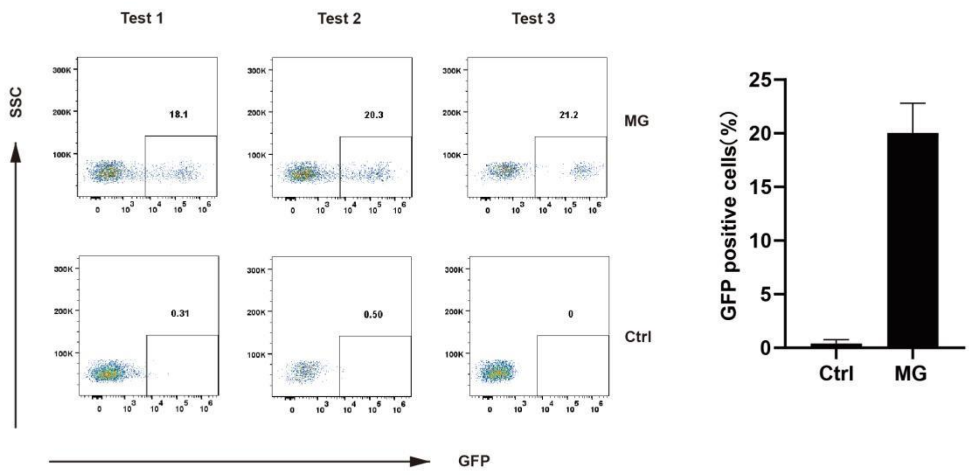

Supplementary Figures S4. High transposition activity of MG in T cells.

Transposition assay demonstrating the activity of MG delivered using an EGFP expression plasmid in T cells. The control groups (Ctrl) lacked the MG transposase plasmid and was filled with hyPB transposase. Data are shown as mean values; error bars, SD. (n=3 independent experiments with 3 different T cell donors).

Figure S5

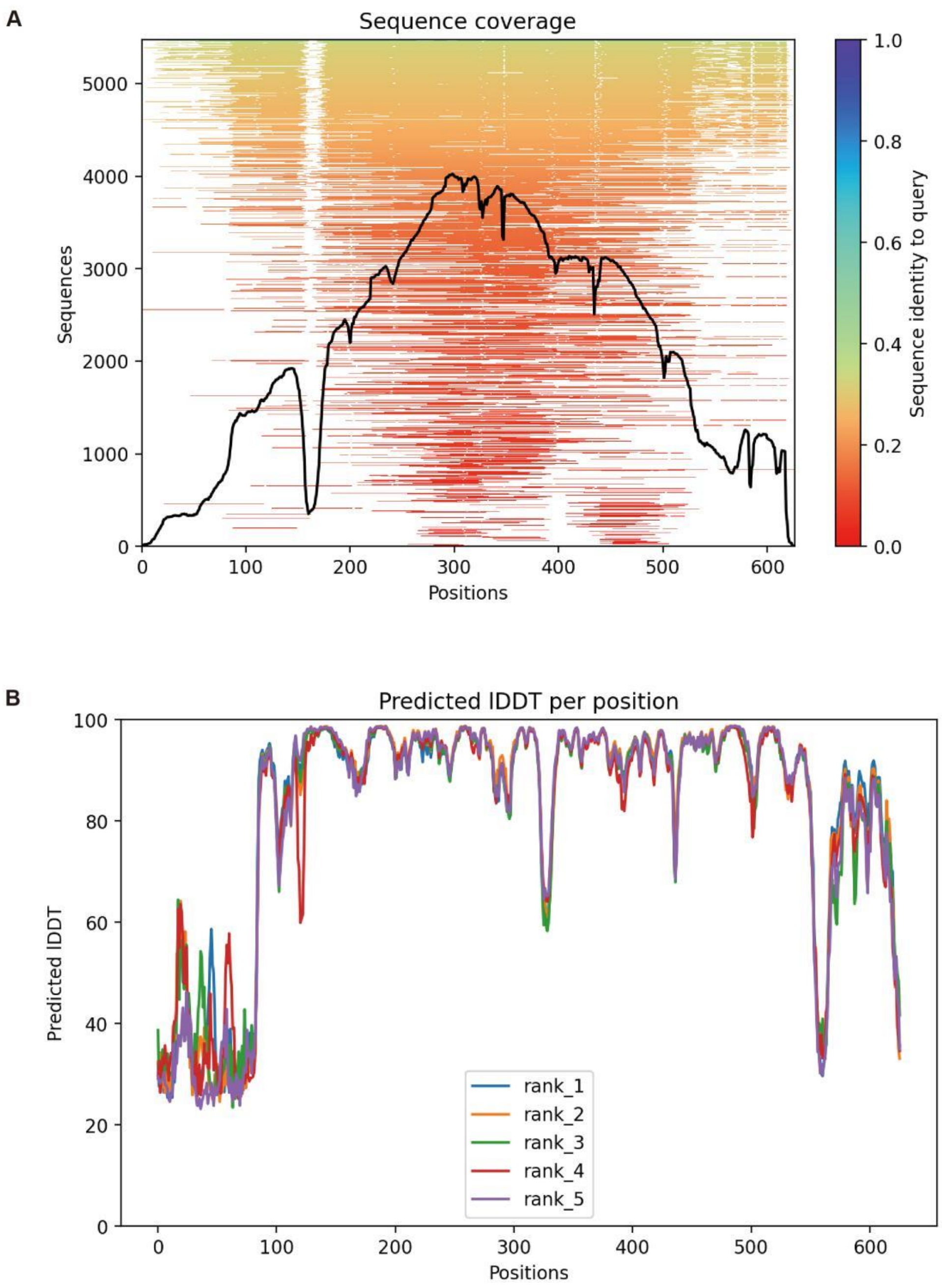

Supplementary Figures S5. AlphaFold data showing the sequence coverage and pLDDT.

(A) Number of identified homologous sequences per position. (B) Confidence scores (lDDT) for the top five predicted models. The lDDT for each residue is presented, with higher values indicating better prediction scores.

Figure S6

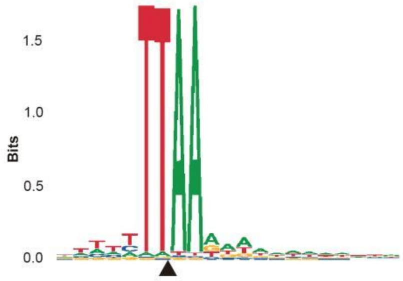

Supplementary Figures S6. The sequence logo of hyMG.

The WebLogo diagrams depict common sequences associated with hyMG transposon insertions. The x-axis represents the upstream and downstream regions surrounding the vector integration site. The height of the letters indicates the level of base conservation, while the y-axis indicates a bit of information. The insertion position is indicated by the black triangle.

Figure S7

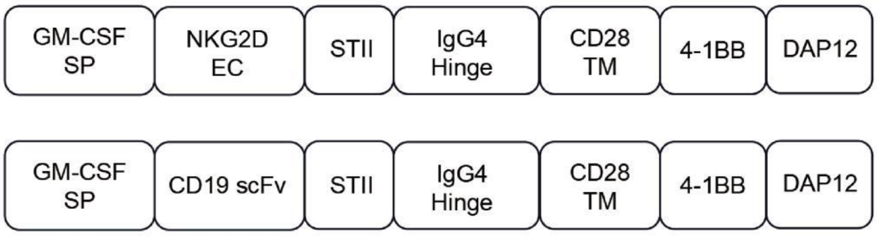

Supplementary Figures S7. NKG2D CAR structural overview.

The 2nd-Gen CAR construct consists of the 4-1BB costimulatory domain and DAP12. Conversely, the control CAR is made up of an anti-CD19 scFv in place of the NKG2D ectodomain. The presence of CAR is identifiable through the Strep-Tag II (STII) tag.

Figure S8

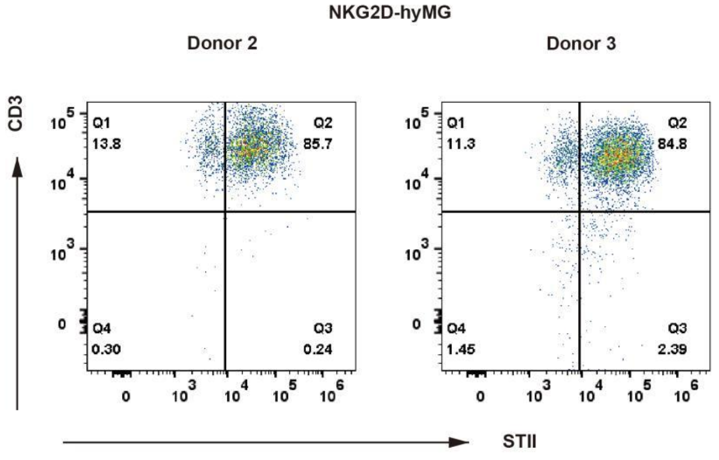

Supplementary Figures S8. Flow analysis of NKG2D generated by hyMG

The phenotype of NKG2D CAR-positive cells generated with hyMG after expansion with irradiated K562A feeder cells. The chart presents the flow analysis results of two additional T cell donors.
